# A Review of the Predictive Role of Plasma D-Lactate Level in Acute Appendicitis: A Myth or Truth?

**DOI:** 10.5402/2011/702372

**Published:** 2011-10-12

**Authors:** Pinar Unverir, Ozgur Karcioglu

**Affiliations:** ^1^Department of Emergency Medicine, Ozel Ege Saglik Hospital, Universal Hospitals Group, Alsancak, 35220 Izmir, Turkey; ^2^Department of Emergency Medicine, Acibadem University, 34140 Istanbul, Turkey

## Abstract

Acute appendicitis (AA) is a common condition which warrants emergency surgery. Detailed history, physical exam, and laboratory findings are often nonspecific in suspected patients. There is substantial evidence to indicate that plasma levels of D-lactate were useful to establish a diagnosis of AA in the medical literature. It has been suggested that it is useful for patients with abdominal pain, especially patients with perforated AA. This paper is designed to highlight the value of D-lactate biomarker in establishing a diagnosis of AA. Based on the literature, it is not helpful for a decision of operation in patients with AA. According to the results of the studies, laboratory involvement was observed between plasma D-lactate level and the final diagnosis of AA, particularly in perforated appendices. It can be considered for routine use in patients with undifferentiated abdominal pain in the emergency department setting.

## 1. Background

Acute appendicitis (AA) is a common condition which warrants emergency surgery. This disease can occur at any age. It is affecting almost 7 percent of the American population, with an incidence of 1.1 cases per 1000 people per year [1]. Appendicitis typically results from luminal obstruction by hyperplastic lymphoid tissue, commonly seen in younger patients, or an appendiceal fecalith, as demonstrated in 1939 by Wangensteen and Dennis [2]. Loss of mucosal integrity and invasion of colonic bacteria across the bowel wall over a few hours are the main pathological mechanisms. The oedema and inflammation may result in vascular compromise, and consequently a small infarct. Perforation is seen in a few days with rupture of the ischemic appendiceal wall. As a rule, the contamination is contained by bowel loops and the omentum to form a phlegmon and, in some cases, an abscess. Manifest peritonitis ensues mostly in children and is commonly associated by the septic shock syndrome.

Most common symptoms of AA are severe epigastric or periumbilical pain, nausea, vomiting, and anorexia. The severity of the symptoms prompts the majority of patients' admissions to the emergency departments (ED) at an early phase of the disease. The classical history of anorexia and periumbilical pain followed by nausea, vomiting, and right lower quadrant pain is noted in only 50% of cases [1]. 

Differential diagnosis of AA comprises a long list of entities. Some diagnoses to distinguish from AA are self-limited causes of abdominal pain not requiring rapid surgical treatment: gastroenteritis, mittelschmerz, ruptured corpus luteum, salpingitis, and mesenteric adenitis. On the other hand, the clinician should have other abdominal processes that warrant surgical intervention in mind; such as perforated ulcer, acute cholecystitis, Meckel's diverticulitis, diverticulitis coli, perforated colon cancer, ruptured ectopic pregnancy, ruptured tubo-ovarian abscess, and ovarian torsion.

Complications include wound infection, peritonitis, perforation, abscess formation, urinary tract disorders, pylephlebitis, and organ failure in untreated patients [1, 3]. The clinical course is mortal in half of the patients without surgery or antibiotics.

## 2. Laboratory Adjuncts in the Diagnosis of Acute Appendicitis

Although most data are elicited via an elaborate history and physical examination, some laboratory and radiological studies can still be useful in the differential diagnosis in patients with acute abdominal pain or “suspect” AA. Laboratory tests should be evaluated together with patient's clinical status and radiological interpretation to establish the accurate diagnosis. Decision-making process for the diagnostic biomarkers of the patients with possible AA remains challenging.

Findings on history, physical examination, and laboratory studies are often nonspecific and insufficient in suspected patients. No single sign, symptom, or diagnostic biomarker accurately confirms the diagnosis of AA. It is known that abdominal computed tomography is more superior diagnostic modality when compared to the laboratory studies to establish a diagnosis in these patients. However, it is still obscure which laboratory biomarker is excellent in pediatric or adult patients before computed tomography or other radiological investigations. Delayed diagnosis represents not only waste of time for the patients and emergency physicians, but also an untoward continuum of suffering. Achievement of a final diagnosis can sometimes be very difficult in the overcrowded EDs.

Average length of stay in the EDs may take more than 3 to 4 hours to reach a proper diagnosis in patients with acute abdomen. Diagnosis in some patients take longer because of multifactorial problems in overcrowded emergencies. In context of the blood chemistry, white blood cell (WBC) count, C-reactive protein (CRP), urine analysis, and beta-human chorionic gonadotropin (beta-hCG) hormone are frequently ordered, depending on the patients' situation [1, 4].

Additionally, some biomarkers most frequently investigated in this context are cytokines, adhesion molecules, interleukins-especially interleukin-6, procalcitonin (PCT), urinary leucine-rich alpha-2-glycoprotein (LRG), S100A8/A9, high mobility group box-1 protein (HMGB1), and D-lactate [5–14].

**WBC**

The WBC count may affect the estimated probability of AA, although not superior to any other diagnostic finding [4, 15]. Marchand et al. pointed out that the positive predictive value (PPV) and negative predictive value (NPV) of an elevated WBC in AA are 92 and 50 percent, respectively [16]. Traditionally, patients are expected to have WBC counts between 12,000 and 18,000 per mm^3^ with AA and 15,000 to 25,000 with perforated appendicitis. WBC counts, however, can be normal or even low and can be confusing if given much weight. Surgeons tend to use the WBC count to make decisions in less than 5% of cases. The WBC count did not significantly influence surgical decision making in the group of patients who were suspected of having AA [17]. An increased percentage of neutrophils (over 75%) and a shift to immature band forms are also typical, but not pathognomonic. These findings represent a bodily response to bacterial infection, not to AA per se, and are also associated with a myriad of conditions.

**CRP**

CRP is also referred to as a nonspecific inflammatory mediator. Many studies have also found in the literature on this subject. Jaye and Waites cited that mixed results had been yielded on the diagnostic value of CRP and erythrocyte sedimentation rate (ESR) in patients with suspected AA [18]. In a recent study, Kwan and Nager pointed out that the diagnosis may be established with high specificity using a combination of CRP and WBC levels in children [19].

**D-Lactate**

Recent studies have shown that elevated concentrations of plasma levels of D-lactate were found to be associated with a diagnosis of AA. D-Lactate is normally present in the blood of mammals at nanomolar concentrations due to methylglyoxal metabolism [20]. D-lactate is a product of anaerobic metabolism and thus released in increased amounts during hypoxia. Many bacterial pathogens produce D-lactate under anaerobic conditions that resemble those found at sites of infection [21].

This biomarker is known to be increased in gastrointestinal diseases particularly intestinal ischemia and gastrointestinal infections. Several reports suggested that D-lactate is considered a highly specific and sensitive test for bacterial infections especially due to intestinal production [22–28]. Continued mucoid accumulation in the obstructed appendix leads to a rise in luminal pressure that causes collapse of draining veins. Ischemic injury in the wall of the appendix causes bacterial proliferation and inflammatory oedema. For that reason, elevation of D-lactate level is not surprising in the patients with AA. Studies also demonstrated high levels of D-lactate in patients with AA in the literature. Based on these facts, we aimed to investigate the relationship of the D-lactate and inflammation of the appendix. Should a positive correlation be detected between D-lactate and AA, it would be very useful for the diagnostic process in the ED setting. This paper is designed to highlight the value of D-lactate biomarker for diagnosis of AA in patients with undifferentiated acute abdominal pain.

## 3. Studies on the Power of D-Lactate as a Diagnostic Biomarker

Many authors have emphasized that diagnosis of the patients with acute abdominal pain is difficult in the EDs. Because of the difficulty of diagnosis of AA, it is still one of the most obscure and, therefore, feared entities in the medicine. There are a lot of research about potential adjuncts in establishing the diagnosis of AA in the literature. In one of the recent studies, Kwan and Nager have evaluated certain biomarkers—CRP, PCT, WBC count, and D-lactate—to distinguish AA from other diagnoses in 209 children with abdominal pain [19]. One hundred fifteen (55%) of them were histologically diagnosed with AA; while 94 served as controls. Mean values of WBC, CRP, and PCT and absolute neutrophil count in subjects with definitive AA were significantly higher than others. D-Lactate levels were found noncorrelative with AA. Unlike other studies on this subject, Kwan and Nager have shown that D-lactate levels is not useful in establishing the diagnosis of AA in pediatric EDs. 

Caglayan et al. conducted a research on preoperative levels of D-lactate, CRP, and WBC counts in 53 children with AA and in 20 healthy controls [29]. It is emphasized in the study that D-lactate, CRP, and WBC counts in the AA patients were significantly higher than in the control group. Based on their data, plasma D-lactate, which had the lowest false negative rate among these laboratory adjuncts, was demonstrated to be a useful diagnostic marker for pediatric patients with suspected AA.

In a different pediatric study by Demircan and colleagues, it has been shown that plasma D-lactate level is useful for perforated type of AA [11]. In this study, D-lactate values were measured in 44 pediatric patients including 23 with AA and 21 with perforated appendicitis before laparotomy. Patients with perforated appendicitis had higher D-lactate levels than patients in the control group and patients with AA. The study suggests that the measurement of plasma D-lactate levels is a useful adjunct to clinical and radiological findings in distinguishing perforated from nonperforated appendicitis in children. According to the study, higher D-lactate levels were distinctive in patients with perforated type of AA than the patients with nonperforated AA. It can be concluded that plasma D-lactate values will be reliable and practicable biomarker for AA disease depending on the Demircan's study.

In a study by Vahl et al., plasma D-lactate levels were recorded in adult patients with acute abdomen at ED [12]. Two hundred patients were included in this study, while those with suspected AA were excluded. The authors researched ESR, WBC, haemoglobin, creatinine, amylase, and D-lactate in patients with acute abdomen. One hundred twenty-eight (64%) correctly received conservative treatment, 54 (27%) of them were operated on within 24 hours, 12 (6%) were correctly treated conservatively and subsequently operated after longer than 24 hours, and 6 (3%) of them were incorrectly treated conservatively within 24 hours. The mean plasma D-lactate and ESR concentrations were higher in the operated patients than in those treated conservatively. The sensitivities of these biomarkers were 75% and 40%, respectively. The D-lactate concentration was increased in 50% of the patients who had not undergone acute surgery following incorrect decisions. Neither plasma D-lactate concentration nor the other standard laboratory tests were observed to have higher predictive value for the determination of indication for surgery. In conclusion, neither plasma D-lactate nor the other laboratory tests were found more accurate in predicting the indication of surgery than clinical examination combined with standard laboratory tests and supplementary radiology. According to this letter, the concentration of D-lactate levels or other laboratory tests in acute abdomen patients are not useful in making the decision of surgical indication. Even if D-lactate is inefficient for the decision of surgery, establishing a proper diagnosis is the most important clinical point to patients with undifferentiated abdominal pain in the EDs. The decision of the operation is to be taken into consideration after proper diagnosis has been established. Expedient diagnosis and emergency surgery are vital in patients with abdominal pain. Early diagnosis of the patients shows the success of the EDs. At the same time, early diagnosis efforts might help to reduce overcrowding in the EDs.

Another study by Duzgun et al. investigated the relation of the D-lactate levels and AA in adults [13]. They evaluated the sensitivity, specificity, PPV, and NPV of the plasma D-lactate levels as a marker for the diagnosis of AA in 32 consecutive subjects. Patient characteristics, ultrasonography, and laboratory variables including WBC, CRP, and D-lactate and intraoperative findings, histology results, and clinical outcome were evaluated. They observed that when the D-lactate level was greater than 0.25 mmol/L in AA, the specificity was 60%, the false negative rate was 25%, and the accuracy was 90%. The false negative rate of CRP (67%) was higher than that of D-lactate levels (25%). Positive correlations were found between D-lactate levels and AA disease. Furthermore, plasma D-lactate had the lowest false negative rate among the other variables. The authors concluded that plasma D-lactate levels might be simple and reliable diagnostic biomarker for adult AA. Although the sample is smaller than the similar studies, the results of Duzgun's study is promising for the EDs. Well-designed, broader studies are pending to draw firm conclusions on the actual value of D-lactate in identification of AA in those with acute abdominal pain.

Similar study by Filiz and colleagues was investigated in Turkey [30]. This study has shown that elevation of D-lactate is associated with early diagnosis of appendicitis. Eighty consecutive patients were prospectively included in this study. The patients were divided into four groups: AA (group 1), perforated AA (group 2), nonspecific abdominal pain (group 3), and acute abdomen other than AA (group 4). For the control group, blood samples were taken in the same manner from 20 healthy subjects. According to the study, there was no significant difference in plasma D-lactate levels between groups 1 and group 2. The plasma D-lactate levels in groups 1 and 2 were significantly higher than those in groups 3 and 4, and the control group. The reliability of D-lactate was calculated as 97% sensitivity, 93% specificity, 90% PPV and 95% NPV, and 95% accuracy. Based on results, plasma D-lactate level may be a valuable biomarker for the diagnosis of AA. Eventually, it was announced that D-lactate level is useful biomarker for the diagnosis of AA. Similar results were also seen in the study of Demircan and colleagues [11]. These studies show the correlation between elevation of the D-lactate and the detection of perforated AA. This biomarker can be used routinely in patients with abdominal pain in the EDs. 

Additionally, Kavakli et al. conducted a study on the diagnostic value of some laboratory variables on preoperative AA diagnosis [31]. Thirty-six consecutive patients with histopathologically confirmed AA were retrospectively included in the study while 15 volunteers were included as control group. Patient characteristics, preoperative ultrasonography, and laboratory assessment including WBC, CRP, and D-lactate levels values were collected. Area under receiver operator characteristic curves for D-lactate was found significant and the cut-off value was found to be 8 mg/dL. Specificity, sensitivity, PPV and NPV calculated for D-lactate were 53%, 80%, 77%, and 57%, respectively. Elevation of D-lactate level's specificity was 53%, sensitivity was 80%, PPV was 77%, and NPV was 57%. Increased D-lactate level is a diagnostic finding for diagnosis of AA according to their research outputs. Studies on this subject in the medical literature are summarized in Table 1.

## 4. Conclusion

It is known that increased levels of D-lactate are associated with bacterial proliferation and ischemic injury in the abdominal pathologies. There is substantial evidence to indicate that D-lactate was used for diagnosis of AA in the medical literature. D-lactate's sensitivity was found between 60 and 96% in patients with AA in the various studies. Although it is not useful in making the decision of the operation, elevation of this biomarker have suggested that it will be a useful indicator for abdominal pain in patients with AA and especially perforated appendicitis. Because the time is an important factor in overcrowded EDs, this biomarker appears to be useful in diagnosis of AA. According to the results of the studies, a positive correlation was observed between plasma D-lactate level and AA. It can be used routinely in the patients with abdominal pain in the EDs by the emergency physicians. However, since plasma D-Lactate is not a specific biomarker for AA, there is still need complementary biomarkers for the diagnosis of the entity. 

## Figures and Tables

**Table 1:** 

Study feature	Kwan and Nager	Caglayan et al.	Demircan et al.	Vahl et al.	Duzgun et al.	Filiz et al.	Kavakli et al.
Setting	Pediatrics	Pediatric surgery	Pediatric surgery	Surgery	Surgery	Surgery	ED
Subject group	Pediatric	Pediatric	Pediatric	Adult	Adult	Adult	Adult
Design	Prospective, observational	Prospective	Prospective	Cross-sectional study	Prospective	Prospective	Retrospective
No. of subjects	230	53	44	200	32	80	36
Measured laboratory parametres	WBC, D-lactate, CRP, PCT, absolute neutrophil count	WBC, D-lactate, CRP	D-lactate	WBC, D-lactate, ESR, amylase, haemoglobin, creatinine	WBC, D-lactate, CRP	D-lactate	WBC, D-lactate, CRP
Major outcome variable	Laboratory tests in patients with abdominal pain suspicious for AA	D-lactate levels in patients with undergoing surgery for AA.	D-lactate levels in identifying the type of AA.	Laboratory tests for the surgical decision.	D-lactate levels in the diagnosis of AA.	D-lactate levels in the early diagnosis of AA.	Laboratory tests in accuracy of preoperative AA diagnosis.
Results	Mean values of WBC, CRP, PCT, and absolute neutrophil count in patients with definitive AA were significantly higher than in subjects with no definitive AA. D-Lactate levels were noncorrelative.	Levels of the D-lactate, CRP, and leukocyte counts in the surgical patients were significantly higher than the healthy patients.	Patients with perforated appendicitis had higher D-lactate levels (3.970 +/− 0.687 mg/dL) than patients in the control group (0.478 +/− 0.149 mg/dL) and patients with AA (1.409 +/− 0.324 mg/dL; *P* < 0.05).	The mean plasma D-lactate concentration and ESR were statistically significantly more often increased in the operated patients than in those treated conservatively.	D-lactate level was greater than 0.25 mmol/L in AA, the specificity was 60%, the false negative rate was 25%, and the accuracy was 90%.	The plasma D-lactate levels in groups 1 and 2 were significantly higher than those in groups 3 and 4 and the control group (*P* < 0.001).	Specificity, sensitivity, PPV, and NPV calculated for D-lactate were as follows: 53%, 80%, 77%, and 57%.
Conclusion	CRP with WBC is useful in distinguishing AA from other diagnoses in the patients. D-Lactate is not a useful laboratory adjunct.	D-lactate may be a useful diagnostic marker for AA.	D-lactat levels may be a useful adjunct to clinical and radiological findings in distinguishing perforated from acute nonperforated AA.	Neither D-lactate concentration nor standard laboratory tests in acute abdomen patients resulted in a better sensitivity for the determination of an indication for acute surgery.	Serum D-lactate had the lowest false negative rate among the other parameters. D-lactate might be a simple and reliable diagnostic marker for AA.	Plasma D-lactate level may be a valuable diagnostic marker for the diagnosis of AA.	Increased D-lactate levels as well as other parameters should be considered as a diagnostic parameter in diagnosis of AA.
Summary and interpretation	D-lactate is not useful in the diagnosis of AA.	D-lactate is useful in the diagnosis of AA.	D-lactate is useful in the diagnosis of AA.	D-lactate is useful in the diagnosis of AA.	D-lactate is useful in the diagnosis of AA.	D-lactate is useful in the diagnosis of AA.	D-lactate is useful in the diagnosis of AA.
